# Placental Villous Explant Culture 2.0: Flow Culture Allows Studies Closer to the In Vivo Situation

**DOI:** 10.3390/ijms22147464

**Published:** 2021-07-12

**Authors:** Nadja Kupper, Elisabeth Pritz, Monika Siwetz, Jacqueline Guettler, Berthold Huppertz

**Affiliations:** Division of Cell Biology, Histology and Embryology, Gottfried Schatz Research Center, Medical University of Graz, 8010 Graz, Austria; nadja.kupper@medunigraz.at (N.K.); elisabeth.pritz@medunigraz.at (E.P.); monika.siwetz@medunigraz.at (M.S.); jacqueline.serbin@medunigraz.at (J.G.)

**Keywords:** flow culture system, placenta, explant culture under flow

## Abstract

During pregnancy, freely floating placental villi are adapted to fluid shear stress due to placental perfusion with maternal plasma and blood. In vitro culture of placental villous explants is widely performed under static conditions, hoping the conditions may represent the in utero environment. However, static placental villous explant culture dramatically differs from the in vivo situation. Thus, we established a flow culture system for placental villous explants and compared commonly used static cultured tissue to flow cultured tissue using transmission and scanning electron microscopy, immunohistochemistry, and lactate dehydrogenase (LDH) and human chorionic gonadotropin (hCG) measurements. The data revealed a better structural and biochemical integrity of flow cultured tissue compared to static cultured tissue. Thus, this new flow system can be used to simulate the blood flow from the mother to the placenta and back in the most native-like in vitro system so far and thus can enable novel study designs.

## 1. Introduction

As a fetal organ, the placenta is temporarily present during pregnancy and serves as the lungs, liver, kidney, and gut of the fetus [1]. The chorionic villi that enable exchange between mother and fetus are organized as villous trees and are freely floating in maternal plasma and blood [2]. The freely floating villi also release a bulk of substances including vesicles, hormones, and growth factors that modulate maternal and fetal physiology [1,2,3].

In vitro analysis of the placenta partly allows examination of its function, regulatory repertoire, and the feto–maternal interface [1,4]. Already in the 1960s, villous explant culture was a prominent approach for transport studies [4,5]. More recently, villous explant cultures were used to analyze placental hormones and factors released into the maternal circulation [6,7]. Although the cultivation of placental explants has been adapted and improved in terms of oxygen concentrations [8,9], the static culture method on the bottom of plastic wells is still the most commonly used approach [4,10]. A variety of static culturing conditions have been developed according to the study design including cultures on the bottom of a well, on a supportive mesh, in a shaking water bath, or freely floating hanging from a styrene block into the medium [4,11,12,13]. However, all these placental explant culture approaches are static methods with no flow around the villi and thus, all of these approaches differ dramatically from the in vivo situation.

Looking at the in vivo situation, placental villi only survive in a floating environment. Already during the first trimester of pregnancy, a first flow of maternal blood plasma traverses from invaded and plugged spiral arteries [14] through the intervillous space and back into invaded uterine veins [15,16]. After dissolution of the arterial trophoblast plugs, at the beginning of the second trimester, maternal blood enters the placenta through spiral arteries. Perfusion of the intervillous space allows maternal blood to flow around placental villi, allowing the exchange of nutrients and oxygen between mother and fetus [2,17,18].

The invasion of endoarterial trophoblasts into spiral arteries results in the dilation of the very end of the vessels [17]. This conversion is important for the subsequent blood flow into the placenta as it results in a reduced blood flow velocity into the intervillous space from 2–3 m/s inside normal arteries to 0.1 m/s within the intervillous space [2,17,18]. Hence, in vivo placental villi are used to a slight fluid shear stress from the beginning of placental perfusion, with plasma during the first trimester, which is followed by blood flow until delivery. This fits to culture experiments indicating beneficial effects of fluid shear stress on the syncytiotrophoblast [19].

Here, we argue that in vitro culture of villous explants should take place in the most functional and native way possible to get robust results representative of the in utero environment. Therefore, this study aimed at establishing a flow culture system under normal placental oxygen conditions for placental villous explants to simulate the blood flow from the mother to the placenta and back in the most native-like in vitro system so far.

## 2. Results

### 2.1. Establishment of the Flow System

**Figure 1:**
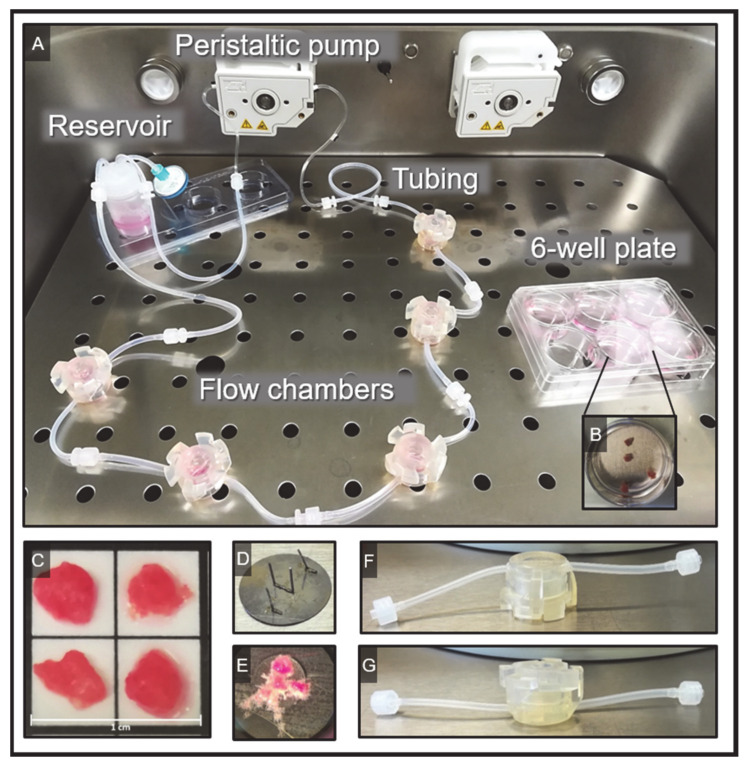
Depiction of the experimental setup. (**A**) The inside of the bioreactor is shown where temperature and gases are controlled. On the left side of the bioreactor a complete flow cycle is assembled showing the reservoir, the tubing, and the flow chambers as well as the peristaltic pump. On the right side, a six-well plate is used as a static control. (**B**) The four explants in a static well are shown. (**C**) Placental villous explants with a cross-sectional diameter of about 0.5 cm are used for the flow and the static explant culture. (**D**) To prevent sweeping away of the explants in the flow-cycle, a metal plate with needles is used to fix the placental explants. (**E**) Then, the metal plate with fixed placental villous explants is introduced into the flow chambers. (**F**,**G**) The flow chambers are used upside-down (**G**) to facilitate the direct exposure of explants to the stream of the medium.

The closed flow system consists of five chambers connected in series with four placental explants per chamber (Figure 1). To prevent the floating of villous explants from the chambers into the tubes, the villi were secured on the bottom of the chamber using small metal plates with needles (Figure 1D,E). To mimic the physiological oxygen concentration in the placenta during the 3rd trimester of pregnancy, O_2_ saturation within the bioreactor was set to 8% and then verified in the medium. The analysis revealed an O_2_ saturation of 8% within the medium after 18.9 min of circulating in the flow system at a flow rate of 1 mL/min (experimental settings are summarized in Supplemental Table S1).

### 2.2. Morphological Analysis

#### 2.2.1. β-Actin

**Figure 2:**
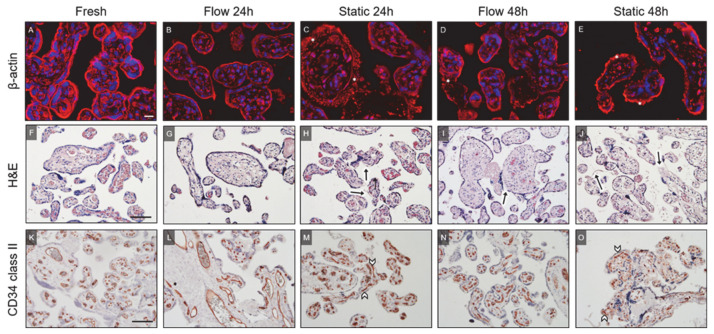
Representative immunofluorescence and (immuno-)histochemical staining of placental villous tissues. (**A**–**E**) The actin cytoskeleton was stained in fresh as well as flow and static cultured tissues using an anti β-actin antibody. (**A**) In fresh tissue, a structured appearance of the cytoskeleton was observed. (**B**–**E**) Cultivation mode and time-depended degeneration of tissue was observed in explant culture. (**C,E**) Asterisks designate increased accumulation of actin microfilaments indicating disintegration of the actin cytoskeleton in static culture. (**A**–**E**) Scale bar represents 20 μm. Six random spots were photographed per slide and used for analysis. (**F**–**J**) H&E staining of placental villi. (**F**) Freshly dissected explants show structured morphology of villi with a dense stroma and noticeable vessels and capillaries. (**F**,**G**) In fresh and flow cultured tissue for 24 h, the syncytiotrophoblast was attached to the stroma, indicating a healthy morphology of the explants. (**H**,**J**) Under static conditions, placental villi appeared partly damaged with augment parts of detached syncytiotrophoblast. (**I**,**J**) The stroma of flow cultured tissue for 48 h (**I**) appeared dense compared to the loose and porous appearance of the stroma in static cultured tissue for 48 h (**J**). Arrows indicate detached syncytiotrophoblast. (**F**–**J**) Scale bar represents 100 μm. (**K**–**O**) CD34 class II staining was applied to visualize feto-placental endothelial cells. (**K**,**L**) Fresh tissue and flow cultured tissue for 24 h show clearly defined and normally arranged endothelial cells. (**M**,**O**) Disrupted vessels are found in static cultured tissue for 24 h (**M**), and this collapsed appearance increased after 48 h of static culture (**O**). (**N**) After 48 h of flow culture, vessels still show structural integrity. (**M**,**O**) Arrowheads indicate damaged and collapsed vessels. (**K**–**O**) Scale bar represents 100 μm.

To assess differences in tissue viability and integrity related to the different culture conditions, diverse immunohistochemistry staining protocols were applied. β-actin staining was used to display the actin cytoskeleton of the villous tissues (Figure 2A–E). In fresh tissue, β-actin staining showed the structured appearance of the cytoskeleton in placental villi, especially in villous trophoblast (Figure 2A). A qualitative analysis of β-actin staining revealed a cultivation mode as well as a time-dependent degeneration of tissues. Disintegration of the actin cytoskeleton became obvious by increased accumulation of actin microfilaments upon cultivation time (Figure 2C–E), especially in tissues cultured under static conditions (Figure 2C,E, asterisks). Since a definite answer as to whether the flow or static cultivation contributes to better tissue preservation cannot be stated based on the qualitative assessment of this staining, further immunohistochemical staining, electron microscopic analysis, as well as analysis of biochemical parameters were performed.

#### 2.2.2. H&E Staining

The morphological differences were visualized, among others, using H&E staining of the villous explants (Figure 2F–J). Freshly dissected explants showed a structured morphology of villi with a dense and compact stroma embedding noticeable vessels and capillaries (Figure 2F). The syncytiotrophoblast was attached to the stroma indicating a healthy morphology of the villi prior to culture. In comparison to fresh tissue, the morphology of explants cultured for 24 h in flow or static conditions appeared partly damaged (Figure 2G,H). On a qualitative level, the morphology of flow cultured tissue seemed to show more preserved parts of villi compared to static cultured tissue. Additionally, more parts of the still attached syncytiotrophoblast seemed to appear in flow cultured tissue after 48 h compared to static cultured tissue after 48 h of cultivation (Figure 2I,J, arrows). Tissue cultured under static conditions for 48 h often showed degenerated parts of villi, which were indicated by a loose and porous appearance of the stroma and collapsed vessels (Figure 2J). In summary, a descriptive analysis of H&E staining revealed that the preserved morphology of the flow cultured tissue represents an intermediate state between freshly dissected tissue and static cultured tissue.

#### 2.2.3. CD34 Class II

CD34 class II staining was used to visualize endothelial cells of vessels within villous explants (Figure 2K–O). Freshly dissected explants displayed clearly defined and normally arranged endothelial cells aiding to identify capillaries and larger blood vessels (Figure 2K). In the static cultured explants for 24 h, more damaged and disrupted vessels were found, and this collapsed appearance increased if static cultivation lasted for 48 h (Figure 2M,O, arrowheads). In comparison to the static culture, the appearance of blood vessels within the explants cultured under flow conditions represented a stage close to blood vessels in freshly dissected explants. This was indicated by the presence of still preserved structural integrity of vessel lining after 24 h and 48 h of flow cultivation (Figure 2L,N).

#### 2.2.4. Cytokeratin 7

**Figure 3:**
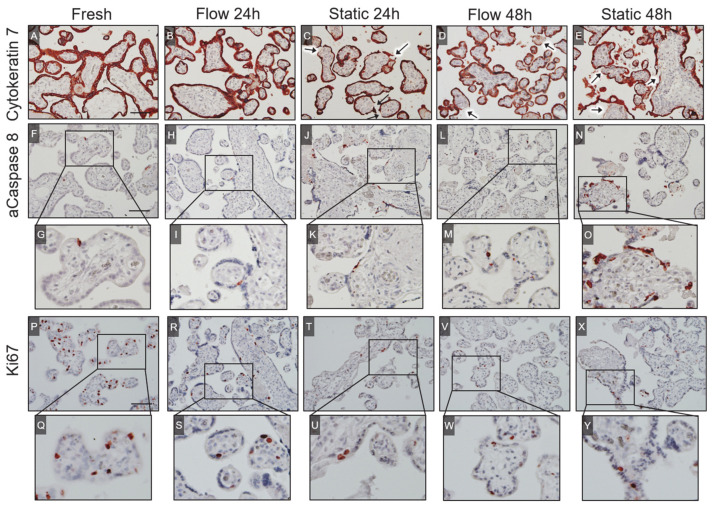
Cytokeratin 7, active caspase 8, and Ki67 staining of villous explants. (**A**–**E**) Cytokeratin 7 staining was used to stain the syncytiotrophoblast as well as villous cytotrophoblasts shown in red. A thick, continuous placental barrier is shown in fresh tissue (**A**) and flow cultured tissue for 24 h (**B**). After 24 h of static culture, the syncytiotrophoblast appeared thinner compared to the fresh tissue (**C**), and detached parts increased after 48 h (**E**). Arrows indicate detached syncytiotrophoblast. (**F**–**O**) Active caspase 8 staining was applied to stain early apoptotic cells in the villous explants, as indicated in red. (**G**–**O**) A zoom in for each image (**F**–**N**) is shown to better display cells with a positive staining for active caspase 8. (**P**–**Y**) Ki67 staining was applied to stain proliferating cells in the tissues, as indicated in red. (**Q**–**Y**) A zoom in for each image (**F**–**N**) is shown to better display cells with a positive staining for Ki67. (**A**–**E**,**F**–**N**,**P**–**X**) The scale bars represent 100 μm.

**Figure 4:**
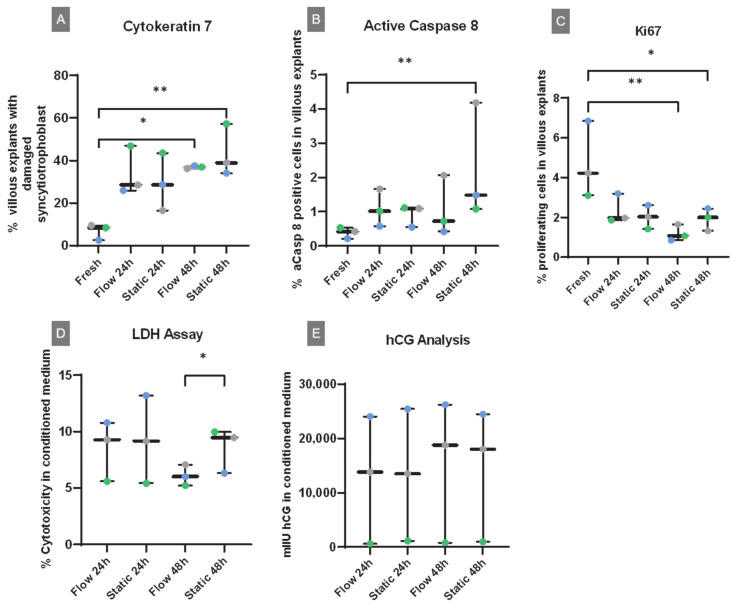
Histological and biochemical integrity and degeneration of placental tissue upon cultivation. For the quantitative analysis of each staining, twelve random spots from the explants were photographed and included for analysis. The median and all data points are shown. The same placenta is color-coded in every condition (gray, blue, green). (**A**) Cytokeratin 7 staining was used to stain villous trophoblast and assess the detachment of this layer from the villous core of villi. There was a significant increase in the amount of detached syncytiotrophoblast in cultured tissues after 48 h in both conditions. The quantification of damaged syncytiotrophoblast around villous explants was performed manually by means of counting villi with detached syncytiotrophoblast versus villi with intact syncytiotrophoblast. (**B**) The quantification of active caspase 8 was performed automatically using the software HALO and counting caspase-positive and caspase-negative cells in the whole tissue area. A small percentage of early apoptotic cells was found in fresh tissues. In tissues cultured under flow conditions, no significant increase of early apoptosis was found, while there was a time-dependent increase in early apoptosis in tissues cultured under static conditions. (**C**) Quantification of Ki67 staining was performed automatically using the software HALO and counting Ki67 positive and Ki67 negative nuclei in the whole tissue area. A high amount of proliferating cells was found in fresh tissues. Significantly decreased levels of proliferating cells were found in flow and static cultured tissue after 48 h of culture. (**D**) An LDH assay was performed to measure necrosis in the tissue cultivated under flow or static conditions. Aside from some variations, there was a significant increase in the necrotic release of LDH in static cultured tissues compared to flow cultured tissues after 48 h of culture. Three independent experiments were used for this analysis, and measurements were done in duplicates. (**E**) Endocrine function was assessed by hCG measurements in the conditioned media. There were no statistically significant differences between the samples. The same three independent experiments as for the LDH assay were used for this analysis. * *p* < 0.05, ** *p* < 0.01.

To visualize the villous trophoblast including the syncytiotrophoblast as well as villous cytotrophoblasts, cytokeratin 7 staining was applied (Figure 3A–E). Intact villi and villi with detached or disrupted syncytiotrophoblast were counted and quantified. Only a small percentage (6.8%) of damaged villi was found in freshly dissected villous explants (Figure 3A and Figure 4A), while there was a significant increase of villi with damaged syncytiotrophoblast after 48 h of culture in both flow and static culture conditions (Figure 3B–E and Figure 4A). The highest number of damaged villi was found in static cultured explants for 48 h (43.5%) (Figure 4A).

#### 2.2.5. Active Caspase 8

Levels of early apoptosis were analyzed using cleaved caspase 8 staining of placental explants cultured in flow or static conditions for 24 h or 48 h and compared to staining levels of freshly dissected explants (Figure 3F–O). Fresh tissue displayed the lowest amount of cleaved caspase 8 positive cells (0.4%) (Figure 4B). There was a 5.8 times higher amount of active caspase 8 positive cells in 48 h static cultured tissue compared to fresh tissue (Figure 4B). No differences were observed in flow cultured tissue after 24 h or 48 h of culture compared to freshly dissected explants (Figure 4B). Although not statistically significant, there seemed to be a trend toward an increase in active caspase 8-positive cells in static cultured explants between 24 and 48 h (Figure 4B). In summary, these data indicate progressive apoptosis in static cultured tissue upon cultivation time, while there is no cultivation time-dependent increase in flow cultured explants (Figure 4B).

#### 2.2.6. Ki67

To stain proliferating cells, an anti-Ki67 antibody was applied on the tissues used in the flow and static experiments as well as for freshly dissected explants (Figure 3P–Y). The highest level of proliferating cells was observed in fresh tissue (Figure 4C). There was a statistically significant decrease of Ki67-positive cells in villous explants between fresh tissue and 48 h cultured tissue in both culturing conditions (Figure 4C). The analysis revealed no significant differences in cell proliferation levels between static and flow cultured tissue for 24 h and 48 h (Figure 4C).

### 2.3. LDH Assay and hCG Measurement

To analyze necrosis in the cultured villous explants, an LDH assay with the conditioned media was performed (Figure 4D). A statistically significant higher cytotoxicity was observed in the media of static cultured explants for 48 h compared to cytotoxicity rates in flow cultured tissue for 48 h (Figure 4D). No further significant differences could be detected. This analysis indicates improved tissue integrity upon longer flow culture compared to static cultured tissue. HCG measurements of the conditioned media were used to analyze the endocrine function of in vitro cultured tissue (Figure 4E). The analysis revealed no statistically significant differences between the samples.

In sum, these data indicate a progressive disintegration of static cultured placental explants with time, while flow cultured tissues occasionally improved or at least maintained their status during the 48 h in vitro culture period.

### 2.4. Ultrastructural Analysis

#### 2.4.1. Scanning Electron Microscopy

**Figure 5:**
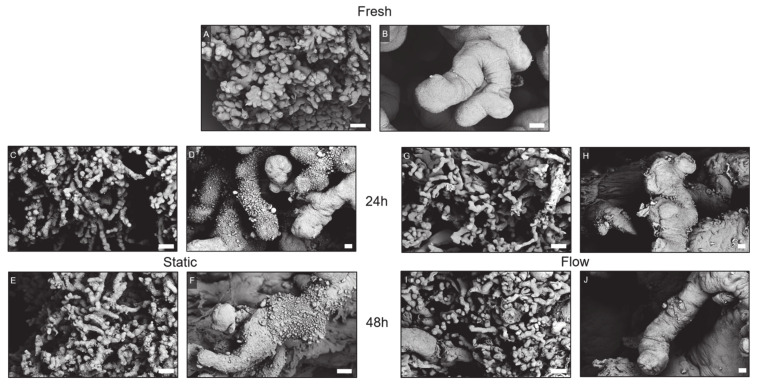
Ultrastructural analysis of representative placental tissue using scanning electron microscopy. (**A**,**B**) Fresh tissue showed a dense microvillous surface accompanied by singular, small vesicular-like structures. (**C**,**D**) After 24 h of static culture, explants revealed a reduction of microvilli and an increased accumulation of vesicular-like structures. (**E**,**F**) After 48 h of static culture, the structural integrity of placental explants dramatically worsened with the further increased presence of accumulated vesicles and an elevated appearance of a stunned, disintegrated microvillous surface. (**G**,**H**) After 24 h of flow culture, the microvillous surface was mostly preserved, while a slightly increased number of vesicular-like structures was observed. (**I**,**J**) After 48 h of flow culture, microvilli were still observed, and the general structural integrity did not seem to worsen between 24 h and 48 h of flow culture. Scale bar represents 100 μm (**A**,**C**,**E**,**G**,**I**), 20 μm (**B**,**F**), or 10 μm (**D**,**H**,**J**).

Scanning electron microscopy was performed to analyze the surface of placental villi in detail (Figure 5A–J). Freshly dissected explants mostly displayed a dense microvillous surface accompanied by occasional singular, small vesicular-like structures (Figure 5A,B). After 24 h of static culture, a clearly reduced number of microvilli was observed, which partially appeared shriveled (Figure 5C,D). Moreover, vesicular-like structures occasionally appeared withered and accumulated. The appearance of the tissue dramatically worsened after 48 h of static culture, as indicated by the increased presence of non-released, accumulated vesicles and the increased appearance of a stunned, disintegrated microvillous surface (Figure 5E,F). The surface of placental explants was also affected after 24 h of flow culture indicated by a slight increase of accumulated vesicular-like structures, mostly in niches between villi. In addition, the number of microvilli on the surface of villi seemed to be slightly reduced (Figure 5G,H). After 48 h of flow culture, the appearance of the tissue did not really change or even worsen compared to the appearance of the explants in the flow culture after 24 h (Figure 5I,J). In flow culture, the villi showed less accumulations of still attached vesicular-like structures, indicating a washing effect of the flowing media similar to the in vivo situation (Figure 5).

#### 2.4.2. Transmission Electron Microscopy

**Figure 6:**
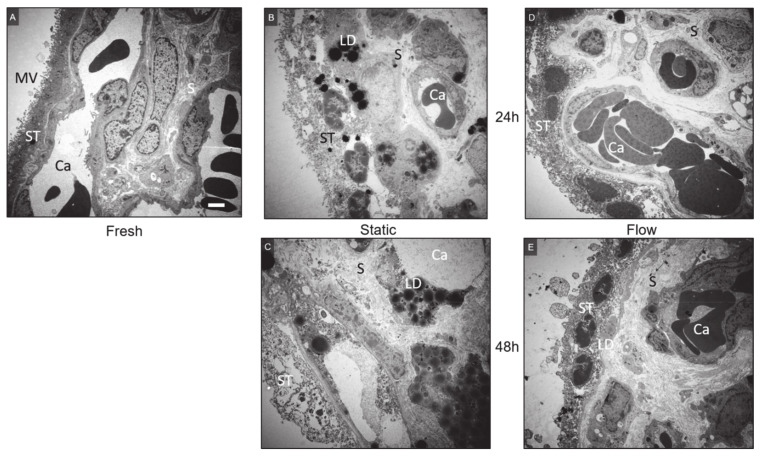
Ultrastructural analysis of representative placental tissue using transmission electron microscopy. (**A**) In fresh tissue, capillaries (Ca) with erythrocytes and endothelial cells were clearly visible. The stroma (S) appeared dense and diverse cells were identifiable. The syncytiotrophoblast (ST) was visible by its dark gray and dense appearance. This layer showed its characteristic appearance of a multinucleated continuous layer with abundant microvilli on the surface and being attached to the basement membrane. (**B**) After 24 h of static culture, the syncytiotrophoblast seemed to detach from the basal membrane and nuclei inside appeared condensed, additionally increased appearance of lipid droplets was identified. (**C**) After 48 h of static culture, accumulation of lipid droplets was found in the cells of the explants. The syncytiotrophoblast was detached from the basement membrane in great extend and appeared loose with hardly identifiable nuclei. Endothelial cells showed vast disintegration. (**D**) After 24 h of flow culture, the tissue occasionally showed parts of detached and loose syncytiotrophoblast. Capillaries and erythrocytes were still clearly visible, and the stroma mostly appeared dense. (**E**) After 48 h of flow culture, the stroma still appeared dense with clearly identifiable capillaries and endothelial cells, while a few lipid droplets appeared in stromal cells. The syncytiotrophoblast was mostly attached to the basal membrane and showed some condensed nuclei inside. Scale bar represents 2 μm. MV: Microvilli, ST: Syncytiotrophoblast, Ca: Capillary, S: Stroma, LD: Lipid droplets.

Transmission electron microscopy was performed to get a deeper insight into the morphology of the cultured tissue (Figure 6A–E). Fresh placental explants showed a structured morphology and no evidence of intracellular vacuoles or edema (Figure 6A). The stroma appeared dense, and diverse cells and organelles were identifiable. Capillaries with erythrocytes, endothelial cells and tight junctions between endothelial cells as well as vascular smooth muscle cells were clearly visible (not shown). Villous cytotrophoblasts were noticeable underneath the syncytiotrophoblast, which was easily identifiable by the more electron-dense cytoplasm (darker gray appearance). The syncytiotrophoblast maintained its characteristic appearance of a multinucleated continuous layer with abundant microvilli on the surface and being attached to the basement membrane (Figure 6A).

After 24 h of static culture, there were obvious changes of tissue morphology. The stroma partially appeared loose and disorganized (Figure 6B). Furthermore, there was an increased appearance of lipid droplets within the cells of the explants, which were only rarely seen in fresh tissues. In concordance to H&E and cytokeratin 7 staining, the syncytiotrophoblast tended to detach from the basement membrane. Additionally, cytotrophoblasts appeared loose and vacuolarized, and nuclei appeared condensed. Moreover, the increased congestion of intravascular erythrocytes and disintegration of endothelial cells was observed (Figure 6B). The degeneration of the tissue worsened after 48 h of static culture indicated by the general vacuolated appearance (Figure 6C). Very large vacuoles within stromal cells and the degenerating syncytiotrophoblast were seen. Microvilli on the syncytial surface were either lost or appeared denuded (Figure 6C).

In comparison to the static cultured tissue, less lipid droplets appeared in the explants cultured under flow (Figure 6D,E). Villous explants cultured under flow for 24 h and 48 h occasionally showed parts of detached and loose syncytiotrophoblast. Erythrocytes and endothelial cells within placental vessels still preserved their normal morphology even after 48 h of flow culture (Figure 6D,E).

In summary, our data indicate that the commonly used static placental explant culture results in the disintegration of placental villous morphology. This effect is diminished through the cultivation of placental explants under flow, especially for long-term cultivation (48 h). The ultrastructural integrity of flow cultured tissue was relatively high; thus, the flow system improved tissue integrity by mimicking the in vivo situation of the blood flow from the mother to the placenta and back.

## 3. Discussion

Diverse systems are available to decipher placental function and its impact on pregnancy pathologies [4,11,13,20]. In vitro culture of isolated primary cells, transfected primary cell lines, or choriocarcinoma cell lines represent useful and simple methods [21]; however, they do not reflect the in vivo micro-environment of cells in a tissue [11]. A prominent and well-established method to look at tissues in vitro is represented by placental dual lobe perfusion [22], which enables the analysis of diverse experimental hypotheses. Although various advantages of this method are known, it is not suitable for experiments with first trimester placentas, and a complete intact organ, including intact blood vessels, is required for the perfusion of the fetal and the maternal side [11,23]. It can also only be used in short-term experiments lasting a couple of hours.

Another commonly used approach is represented by the static in vitro culture of placental explants [4,12]. Different to the in vivo situation where blood is flowing around placental villi, this static method of culturing placental explants finds the explants mostly lying in medium on the bottom of the wells and thus differs dramatically from the in vivo situation. This fact has already been discussed in various studies [11,12,24]. It is also reflected in our data shown here. Watson et al. have already stated the difficulties of placental in vitro organ culture associated with the sensitivity of the syncytiotrophoblast [24], since it represents a terminally differentiated and highly sensitive epithelium [24,25]. Therefore, we established a gentler and still simple method to cultivate placental explants under flow in contrast to the commonly used static approach.

The in vitro flow system for placental explants simulates the perfusion within the intervillous space, thus mimicking an in-utero-like environment. Explants were cultured under flow (1 mL/min, 8% O_2_, 5% CO_2_) for 24 h and 48 h in a closed humidified environment. The upside-down position of the chambers facilitated the direct exposure of explants to the flow of medium. In the normal upside-up position of the chambers, flow only passes on top of the explants without direct exposure of the explants to the flow of the medium.

The flow rate of 1 mL/min was used based on literature research [17,18,26,27,28]. So far, the in vitro culture of trophoblast under flow was performed using single cells including primary term trophoblasts, choriocarcinoma cells, as well as trophoblastic stem cells [27]. The flow rates used in these systems ranged from 2 µL/min to 5 mL/min. Hence, the flow rate used here for villous explants (1 mL/min) was chosen to be in the middle of what has been used so far. In general, maternal blood flow through the intervillous space of the placenta starts with a relatively high velocity (0.1 m/s) [17], is hypothesized to be reduced while passing the villous trees and may then speed up again when it is drained back into uterine veins. Thus, it is debatable whether it is possible to calculate the actual flow rate of blood in the intervillous space for a given placental villus, since this flow rate depends on the position of the individual villus in the intervillous space and its distance to the spiral arteries and the uterine veins.

In our study, the impact of the flow system on placental tissue viability and structural integrity was analyzed using unbiased morphological and biochemical parameters. Previous studies reported superfused placental explants at a moist cellulose filter inserted in a perfusion chamber [29,30,31]; however, they revealed restricted tissue viability [20]. To our knowledge, this study is the first proving the benefits of culturing tissues under flow in terms of biochemical and structural viability. We showed a well-preserved morphology of flow cultured tissue compared to static cultured tissue up to 48 h of in vitro culture. All data showed a higher tendency of tissue disintegration of static cultured tissue compared to flow cultured tissue.

The endocrine function of the explants was assessed by measuring hCG levels in conditioned media from flow and static explant cultures. In healthy pregnancies, hCG levels exponentially rise during the first weeks of gestation and peak at around 10 weeks, which is followed by a slow decline until the end of pregnancy [7,32]. It seems to play a role in placental growth by triggering the fusion and differentiation of the cytotrophoblast with the syncytiotrophoblast [33]. Notably, in our study, there was no statistically significant time-dependent and condition-dependent effect on hCG secretion. The study of Siman et al. showed that when using static conditions, hCG release from cultured explants is increased from the second day onwards [12]. The authors attributed this effect on the regeneration of the syncytiotrophoblast over time; however, they could not exclude increased damage and thus necrotic release of hCG into the media [12].

Electron microscopical imaging data provided further detailed insight into the morphology of the tissue and revealed a cultivation-time dependent degeneration, especially under static culture conditions. Although it should be noticed that transmission and scanning electron microscopical data are primarily descriptive, a progressive degeneration of the syncytiotrophoblast upon static in vitro culture of placental tissue was reported before, and it was observed in first trimester [11,34] as well as third trimester tissues [11,12]. In concordance to our data, these studies showed intracellular vacuoles in the syncytiotrophoblast after 8 h [11] and 1 day [12] in static cultured placental tissue. By contrast, in first trimester explants, Palmer et al. showed a newly formed trophoblast layer already after 48 h of static explant culture [34]. For explants from term placenta, Siman et al. indicated syncytiotrophoblast degeneration over time upon static in vitro culture followed by regeneration beginning at 7 days of in vitro culture [12].

Watson et al. stated that the general deterioration of the microvilli on the surface of static cultured tissue could be symptomatic for the degeneration of the apical membrane of the syncytiotrophoblast [24]. Due to the observed chromatin condensation and membrane blebbing, which are major characteristic features of the apoptosis process [35], the authors suggested that syncytiotrophoblast degeneration in static cultured tissue may be driven by apoptosis [12]. This notion is in line with our ultrastructural analysis of the static cultured tissue as well as confirmed with the quantification of the immunohistochemical staining for active caspase 8, which showed increased apoptosis in static cultured tissue at 48 h.

In addition, the increased appearance of lipid droplets observed after 48 h in static cultured tissue was observed in the study of Palmer et al. [34]. This further indicates increased apoptosis in static cultured tissue, since the induction of apoptosis is associated with an accumulation of cytoplasmic lipid droplets [36]. This observation could represent a trophoblastic defense mechanism protecting the placenta against lipotoxicity [37,38,39]. The study of Bildirici et al. showed increased lipid accumulation in placental villi from pregnancies complicated by fetal growth restriction (FGR) as well as in primary trophoblasts treated with hypoxia (<1% O_2_), indicating hypoxia-induced diminished fatty acid oxidation. The authors supposed a link between increased lipid droplet storage in FGR and placental insufficiency [37]. This notion can be supported by our findings, showing placental tissue damage and increased lipid droplet accumulation after 48 h of static culture. In terms of flow cultured tissue, also a slight degeneration of the syncytiotrophoblast was observed after 24 h; however, the degeneration seemed to attenuate or at least remain at the same level after 48 h of flow culture.

Two new and interesting aspects of the flow cultures need to be mentioned. One is that the endothelial cells of the placental vessels within the explants only remain intact in explants cultured under flow. Although in all explants, there is no flow within the placental vessels, only those with flow on the outside of the villi show cellular integrity over the 48 h culture period, while those cultured under static conditions disintegrate quite soon. The second interesting aspect can be found on the surface of the cultured villi. Under static conditions, there is an accumulation of vesicular structures protruding from the apical membrane of the syncytiotrophoblast. By contrast, there are less such vesicular structures on the villous surface of those explants cultured under flow conditions. Such vesicles remained only in niches where very little flow is expected. Hence, it seems as if these vesicles are detached from the villous surface by means of shear stress. If this is true, then the flow culture would be an ideal culture method to mimic the in vivo situation regarding the release of vesicles from the syncytiotrophoblast into the maternal circulation.

Consequently, the analysis of the feto–maternal interface using placental explants cultured under flow conditions would enable deciphering the etiologies of different pregnancy pathologies, including preeclampsia. A growing body of literature has already elaborated the complexity of many serious and common pregnancy-related diseases such as preeclampsia, fetal growth restriction, and gestational diabetes mellitus. However, insights into the pathophysiology and diagnosis of these syndromes are still missing, which often leads to premature births with all its consequences. In addition, also affected women may have long-term health problems such as risk disposition for cardiovascular diseases [40,41,42,43]. Hence, finding specific diagnostic and curative treatments for the mother and fetus is a demanding task, requiring systemic studies of the placenta and the feto–maternal interface using innovative study designs. One of these new study designs could be provided with this flow system. Indeed, there is more to come in terms of automatic explant sampling as well as approaches for upscaling the quantity of explants per experiment. Nevertheless, to our knowledge, we are the first to cultivate placental explants under constant flow conditions. Furthermore, this approach may be used for first trimester placental explant culture as well and also to simulate diverse conditions of pregnancy by changing the variable conditions. Additionally, introducing endothelial cells directly behind the villous explants in the flow system will enable mimicking the blood flow from the placenta to the mother in the most native-like in vitro system so far.

## 4. Materials and Methods

### 4.1. Human Placental Samples

This study was approved by the ethics committee of the Medical University of Graz (31-019 ex 18/19 version 1.2). Placental tissue from 3rd trimester deliveries between weeks 34 and 40 of uncomplicated pregnancies was used for the study (*n* = 3) with written informed consent from women undergoing C-sections.

### 4.2. General Culture of Villous Explants

Immediately after delivery, samples from three areas around the central region of the placenta were dissected with a size of 2 cm^3^. Chorionic plate, maternal decidua, and areas of visible infarcts were discarded. The remaining villous tissue was further dissected into villous explants with a wet weight of approximately 7.5 mg (about 0.5 cm cross-sectional diameter) and used for explant cultures.

Villous explants were washed with PBS (ThermoFisher Scientific, Waltham, MA, USA) and transferred into pre-warmed medium (PromoCell PC-C-22120, Heidelberg, Germany; without EGCS/h and FCS) and supplemented with 5% exosome-depleted fetal bovine serum (Gibco by Life Technologies, ThermoFisher Scientific, Waltham, MA, USA) and 1% penicillin/streptomycin (Gibco by Life Technologies, ThermoFisher Scientific, Waltham, MA, USA). The tissue was cultured at 37 °C for 24 h or 48 h in a humidified atmosphere containing 8% O_2_ and 5% CO_2_ under static or flow conditions using a flow bioreactor (TEB500, EBERS Medical Technology SL, Zaragoza, Spain). O_2_ saturation in the circulating medium was verified with an external O_2_ measurement device (PreSens, Fibox 3, Regensburg, Germany).

#### Flow and Static Culture of Villous Explants

The flow chambers with a dimension of 23 mm height × 37 mm diameter and 15 mm internal chamber width (Kirkstall Ltd., Quasi Vivo^®^, North Yorkshire, UK; Supplemental Table S2) were filled with 2 mL of PromoCell medium. The chambers were connected with tubes having a diameter of 1/16” ID for the inlet and a diameter of 3/32” ID for the outlet tubes. Since the inlet and outlet tubes from the chamber are positioned on the upper side of the chambers, the chambers were turned upside-down to facilitate the exposure of explants to the direct stream of medium. Four explants were transferred into each chamber and secured inside the chamber with stainless steel pins. One flow cycle consisted of five chambers connected in series with approximately 30 mg of villous tissue per chamber and a total wet weight of all explants of about 150 mg. Using the peristaltic pump system integrated into the TEB500 system and an additional pumping tube of 1.02 mm diameter (Tygon^®^, Bartelt, Graz, Austria), villous explants were perfused with a flow rate of 1 mL/min. The flow system was filled with a total volume of 25 mL. The specifications of the flow system are summarized in Supplemental Table S2. Depiction of the flow system is represented in Figure 1.

For the static culture, placental explants from the same placentas as used for the flow cultures were cultured in 6-well plates (NUNC, ThermoFisher Scientific, Waltham, MA, USA) filled with 4 mL of PromoCell medium and 4 villous explants per well (30 mg villous tissue per well, 150 mg villous tissue per well plate). The static explants were placed in the TEB500 flow bioreactor and cultured in the same humidified atmosphere as the flow culture explants.

### 4.3. Histology and Immunohistochemistry

Explants were fixed in formalin (4%) for up to 48 h followed by paraffin embedding using an Excelsior AS Tissue Processor (ThermoFisher Scientific, Waltham, MA, USA). Five µm sections from formalin-fixed paraffin-embedded tissues (FFPE) (Microtome Microm HM 355 S, ThermoFisher Scientific, Waltham, MA, USA) were mounted on Superfrost Plus slides (Menzel-Glaeser, Braunschweig, Germany). The sections were deparaffinized using Histolab Clear^®^ (Histolab^®^, Askim, Sweden) solution and rehydrated through a graded series of ethanol. For each FFPE sample, a hematoxylin–eosin staining was performed. Antigen retrieval was performed in a microwave oven (40 min, 150 W, Miele, Guetersloh, Germany) using preheated 10 mM Tris EDTA buffer (pH 9) or 10 mM citrate buffer (pH 6).

Immunohistochemistry (IHC) was performed utilizing the UltraVision LP-Detection System HRP-Polymer (ThermoFisher Scientific, Waltham, MA, USA) according to the manufacturer’s instructions. Briefly, endogenous peroxidase was blocked for 10 min with Hydrogen Peroxide Block (ThermoFisher Scientific, Waltham, MA, USA). After three washing steps (Tris-buffered saline with 0.05% Tween, TBST) slides were incubated with UltraVision Protein Block for 7 min (ThermoFisher Scientific, Waltham, MA, USA) and then incubated with the primary antibodies diluted in antibody diluent (Dako, Santa Clara, CA, USA) for 45 min: cytokeratin 7 (1:1000, OVTL 12/30, Invitrogen, Waltham, MA, USA), CD34 Class II (1:500, QBEnd-10, Dako, Santa Clara, CA, USA), cleaved caspase 8 (1:100, clone 18C8, Cell signaling, Danvers, MA, USA), or Ki67 (1:50, clone MIB-1, Dako, Santa Clara, CA, USA). Primary antibody enhancer was applied for 10 min, which was followed by incubation for 15 min with Large Volume HRP Polymer. Then, the slides were incubated with the substrate amino-ethyl carbazole (AEC substrate kit, Abcam, Cambridge, UK) for 10 min. Nuclei were counterstained with Mayer’s Haemalaun for 10 min. After the staining procedure, all slides were mounted with Kaiser’s Glycerin Gelatine (Merck, Darmstadt, Germany) and analyzed with a Leica DM 6000 B microscope (Wetzlar, Germany) equipped with an Olympus DP 72 Camera.

If not stated otherwise, twelve random spots per slide were photographed with 200× magnification by manual rotation of the stage using a joystick and then used for analysis. Semi-quantitative analysis was performed using the HALO software (v3.1.1076.342, indica labs, Albuquerque, NM, USA). For cleaved caspase 8 and Ki67, data are given as percentage of positive cells per total number of cells in the analyzed tissue area. A pipeline of the quantification is depicted in Supplemental Figure S1. Cytokeratin 7 was used to quantify the detachment of villous trophoblast from the villous stromal core. This was achieved by counting villi with detached or disrupted syncytiotrophoblast per total villous count.

For immunofluorescence staining, the slides were washed with PBS and incubated with UV Block (Thermo Fisher Scientific, Waltham, MA, USA). The primary antibody (anti-β-actin, 1:10,000, AC-15, Abcam, Cambridge, UK) was diluted in antibody diluent (Dako, Santa Clara, CA, USA) and incubated on the slide for 30 min. After washing steps, slides were incubated with the secondary antibody for 30 min (Alexa Fluor 555 goat-anti-mouse, 1:200, ThermoFisher Scientific, Waltham, MA, USA), while nuclei were stained with DAPI (1:1000, ThermoFisher Scientific, Waltham, MA, USA) for 5 min. Slides were dried at room temperature in the dark and mounted with ProLong™ Gold Antifade Reagent (ThermoFisher Scientific, Waltham, MA, USA). Pictures were taken with an Olympus microscope (BX3-CBH) (Hamburg, Germany) at 400× magnification. A summary of the used antibodies is shown in Supplemental Table S3.

### 4.4. Ultrastructural Analysis

The fixation and preparation of villous explants for electron microscopy was performed according to standard electron microscopy protocols. In brief, villous explants were fixed in 2% paraformaldehyde/2.5% glutardialdehyde in 0.1 M cacodylate buffer (pH 7.4) for 2 h and then transferred into 0.1 M cacodylate buffer. After post fixation in 2% osmium tetroxide in 0.1 M cacodylate buffer, samples were dehydrated in a graded series of ethanol.

#### 4.4.1. Transmission Electron Microscopy (TEM)

Samples for transmission electron microscopy were transferred into propylene oxide as an intermedium and subsequently embedded in TAAB epoxy resin (Agar Scientific, Stansted, Essex, UK). Ultrathin sections (70 nm) were cut using a Leica UC7 ultramicrotome (Leica Microsystems, Vienna, Austria) and then stained with platinum blue and lead citrate. Images were taken with 80 kV acceleration voltage using a Zeiss EM 900 transmission electron microscope (Zeiss, Oberkochen, Germany).

#### 4.4.2. Scanning Electron Microscopy (SEM)

After dehydration in a graded series of ethanol, samples for scanning electron microscopy were critically point dried (CPD 030; Bal-Tec, Balzers, Liechtenstein) and sputter coated with gold palladium (SCD 500; Bal-Tec, Balzers, Liechtenstein). Images were taken using a Zeiss Sigma 500 field emission scanning electron microscope (Zeiss, Cambridge, UK) with a back-scattered electron detector at 5 kV acceleration voltage.

### 4.5. LDH Assay and hCG Measurement

For LDH and hCG measurements, the conditioned culture media from placental villous explants were used. Prior to performing the LDH assay (LDH Cytotoxicity Detection Kit, Takara, Japan) and the hCG measurement, the medium was centrifuged at 1500× *g* for 10 min, and the supernatants were stored at −80°C. The LDH assay was performed according to the manufacturer’s instructions in 96-well plates (NUNC, ThermoFisher Scientific, Waltham, MA, USA). For measuring the absorbance of the samples at 492 nm with a reference wavelength at 620 nm, a Spark TM 10 M multimode microplate reader (TECAN, Maennedorf, Switzerland) was used. All samples were measured in duplicates. HCG was measured in routine immunoassay analyses at the department of Obstetrics and Gynecology at the Medical University of Graz (Dimension Xpand; Dade Behring Inc., Deerfield, IL, USA). Obtained values were corrected to the primarily used amount of medium in the experiments.

### 4.6. Statistical Analysis

Data were analyzed and visualized using GraphPad Prism 9.0.0. (San Diego, CA, USA). Unless stated otherwise, experiments were performed in triplicate. Statistical differences were calculated by ordinary one-way ANOVA using a nonparametric test (Friedman Test) without multiple comparison. A *p*-value of less than 0.05 was considered statistically significant.

## Data Availability

The data that support the findings of this study are available from the corresponding author upon reasonable request.

## Supplementary Materials

The following are available online at https://www.mdpi.com/article/10.3390/ijms22147464/s1.
