# Supplementary material for: Placental Villous Explant Culture 2.0: Flow Culture Allows Studies Closer to the In Vivo Situation
Source: Int J Mol Sci. 2021 Jul 12;22(14):7464. doi: 10.3390/ijms22147464 (PMC8308011; doi:10.3390/ijms22147464)
Supplement: Supplementary file 1 [file ijms-22-07464-s001.zip › ijms-1293096-supplementary.pdf]

Supplements

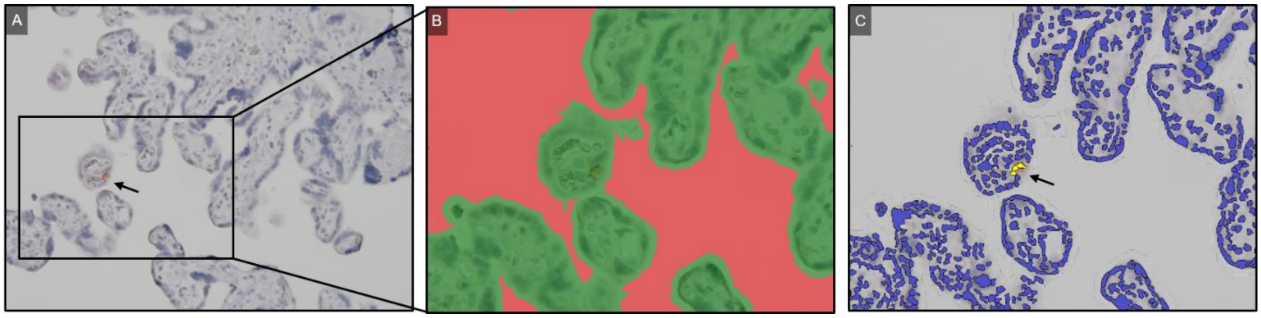

Figure S1: Representative example of the pipeline for the quantitative analysis of active caspase 8 staining using the HALO software. (A) Original picture of fresh placental explants stained for active caspase 8 (red dot, arrow). (B) The software identified the tissue area, marked in green. (C) Then the software identified all nuclei in the tissue area (blue) and marked the active caspase 8 positive cells in yellow (arrow). The analysis revealed 418,624.5 μm^2 tissue area, with 2,118 nuclei in total. Three cells were positive for active caspase 8.

Table S1: Experimental settings used for placental explant flow-culture in the bioreactor Ebers TEB500

**Flow rate**	1 ml/min
**O_2**	8 %
**CO_2**	5 %
**Temperature**	37 °C
**Cultivation period**	24 h and 48 h

Table S2: QV500 specification (Kirkstall Ltd, Quasi Vivo®, North Yorkshire, UK)

**Volume of chamber**	2 ml
**Chamber width**	15 mm internal
**Chamber depth**	10 mm from culture surface to top of chamber base
**Overall dimensions**	23 mm height x 37 mm diameter
**Diameter of tubing**	Inlet: 1/16” ID, Outlet: 3/32” ID

Table S3: Antibodies used for immunohistochemistry and immunofluorescence

Antibody	Original Concen-tration	Dilution	Clone	Species	Isotype	Source
**Cytokeratin 7**	0.2 mg/ml	1:1000	OVTL 12/30	Mouse, mAb	IgG1	Invitrogen
**Ki67**	35 mg/l	1:50	MIB-1	Mouse, mAb	IgG1	Dako
**CD34 Class II**	12mg/l	1:500	QBEnd-10	Mouse, mAb	IgG1/κ	Dako
**Cleaved caspase 8**	91 µg/ml	1:100	18C8	Rabbit, mAb	IgG	Cell signaling
**ß-Actin**	2.1 mg/ml	1:10000	AC-15	Mouse, mAb	IgG1	Abcam
